# 2009-2017 RACIAL AND ETHNIC DISPARITIES IN MEDICARE PATIENT EXPERIENCE AND INFLUENZA IMMUNIZATION

**DOI:** 10.1093/geroni/igz038.1600

**Published:** 2019-11-08

**Authors:** Marc N Elliott, Steven Martino, Katrin Hambarsoomian, Shondelle Wilson-Frederick, Jacob Dembosky, Sarah Gaillot, Robert Weech-Maldonado, Amelia Haviland

**Affiliations:** 5 Carnegie Mellon University, Pittsburgh, Pennsylvania, United States; 2 RAND Corporation, Pittsburgh, Pennsylvania, United States; 3 Centers for Medicare & Medicaid Services, Baltimore, Maryland, United States; 4 University of Alabama at Birmingham, Birmingham, Alabama, United States; 1 RAND Corporation, Santa Monica, California, United States

## Abstract

We sought to understand the extent to which racial and ethnic disparities in the immunization rates and case-mix adjusted patient experiences of access (getting needed care and getting care quickly) of Black, Hispanic, and non-Hispanic White Medicare beneficiaries have changed over time. Accordingly, we analyzed 2009-2017 CAHPS data from 2,725,614 Medicare beneficiaries. In 2009, flu immunization rates for Black and Hispanic beneficiaries were lower than non-Hispanic White beneficiaries by 17 and 14 percentage points, respectively. Over 9 years, these gaps were reduced to 12 and 8 points, respectively (p<.01 for all comparisons). In 2009, Black beneficiaries had 2-point and 5-point disparities on getting needed care and getting care quickly (on a 0-100 scale) respectively, relative to non-Hispanic Whites. For getting needed care, there was no significant change over time in the gap between Blacks and non-Hispanic Whites. For getting care quickly, the gap between Blacks and non-Hispanic Whites narrowed to 3 points in 2017. In 2009, Hispanic beneficiaries had 2-point and 5-point disparities on getting needed care and getting care quickly, respectively, compared to non-Hispanic Whites. The gap on getting needed care widened by 1 point to a 2017 disparity of 3 points. For getting care quickly, there was no significant change over time in the gap between Hispanics and non-Hispanic Whites. These findings suggest that flu immunization rates for Black and Hispanic Medicare beneficiaries have improved significantly relative to non-Hispanic Whites; however, substantial disparities remain. For the patient experience measures, the findings are more mixed.

